# Larval source management in Ethiopia: modelling to assess its effectiveness in curbing malaria surge in dire Dawa and Batu Towns

**DOI:** 10.1186/s12936-024-05189-2

**Published:** 2024-12-03

**Authors:** Galana Mamo Ayana, Abdollah Jalilian, Temesgen Ashine, Eshetu Molla, Elifaged Hailemeskel, Dagmawi Hailu Yemane, Hailegiorgis Yirgu, Nigatu Negash, Natnael Teferi, Daniel Teshome, Alison M. Reynolds, David Weetman, Anne L. Wilson, Birhanu Kenate, Martin J. Donnelly, Luigi Sedda, Endalamaw Gadisa

**Affiliations:** 1 Malaria and Neglected Tropical Disease, Armauer Hansen Research Institute, Addis Ababa, Ethiopia; 2 Lancaster Ecology and Epidemiology Group, Lancaster Medical School, Lancaster University, Lancaster, UK; 3 Public Health Emergency Management, Research, and Blood Bank Service Directorate, Oromia Region Health Bureau, P.O. Box 24341, Addis Ababa, Ethiopia; 4 Public Health Emergency Management, Research, Dire Dawa Region Health Bureau, Dire Dawa, Ethiopia; 5 Department of Vector Biology, Liverpool School of Tropical Medicine, Pembroke Place, Liverpool, L35QA UK

**Keywords:** Malaria, Larval, *Bti*, Source reduction, Interruption, Time series

## Abstract

**Background:**

Ethiopia faces several severe challenges in terms of malaria elimination, including drug resistance and diagnostic evasion in the *Plasmodium falciparum* parasite, insecticide resistance in the primary *Anopheles* malaria vector, and, most recently, the invasion of the Asian malaria vector *Anopheles stephensi*. Novel malaria control methods are therefore needed, and in this paper, we describe the evaluation of a larval source management (LSM) strategy implemented in response to *An. stephensi*. The primary outcome was the malaria incidence rate compared between intervention and non-intervention sites in the presence of *An. stephensi*.

**Methods:**

Intervention (Batu and Dire Dawa) and control (Metehara) towns were selected, and weekly malaria passive case detection data collected between 2014 and 2023 were obtained from the Oromia regional state and Dire Dawa City Administration Health Bureau. In addition, data regarding intervention were obtained from the President’s Malaria Initiative (PMI) reports. Weekly malaria passive case data were used to evaluate the change in the estimated malaria incidence rate and trends of temporal patterns of the estimated malaria incidence rate before and after interventions. An interrupted time series model with a cyclic second-order random walk structure periodic seasonal term was used to assess the impact of LSM on malaria incidence rate in the intervention and control settings.

**Results:**

An upsurge in malaria cases occurred after 2020 at both the intervention and control sites. The temporal patterns of malaria incidence rate showed an increasing trend after the intervention. The ITS model depicted that the LSM has no impact in reducing the malaria incidence rate at both intervention site Dire Dawa [immediate impact = 1.462 (0.891, 2.035)], [Lasting impact = 0.003 (− 0.012, 0.018)], and Batu [Immediate impact 0.007 (− 0.235, 0.249), [Lasting impact = 0.008 (− 0.003, 0.013)].

**Conclusions:**

An overall increasing trend in the malaria incidence rate was observed irrespective of the implementation of LSM in the urban settings of Ethiopia, where *An. stephensi* has been found. Further investigations and validations of the incorporation of LSM into control activities are warranted.

## Background

By scaling up and sustaining malaria control interventions, in 2019, Ethiopia achieved the Global Technical Strategy (GTS) target of a 40% reduction in incidence by 2020 compared to 2015 [1]. The number of cases and deaths decreased by more than 88% and 96.5%, respectively, between 1990 and 2015 [2]. To consolidate the gains made and to drive malaria elimination from the country, the 2021 to 2025 National Malaria Strategic Plan (NMSP) was developed to identify priority areas for evidence-based actions. It is based on the stratification of Ethiopia into high (API > = 50), medium (API > = 10 & < 50), low (API > 5& < 10), very low (API > 0 & < = 5), and malaria-free (API = 0) areas based on the Annual Parasite Incidence (API: cases/ 1000 people/year). On average, the API for Batu, Metehara, and three of the Dire Dawa were 16.60, 77.31, and 70.73 cases/1000 people, respectively. In this strategy, the following priorities are identified: early diagnosis and treatment, empowering and mobilizing the community, enhancing vector control, and improving the system for surveillance and response. Additionally, it encourages the involvement of stakeholders to conduct operational research and carry out monitoring and evaluation (M&E) [3]. The country aimed to achieve zero indigenous malaria cases in 565 districts in 2025 from a total of 810 malaria endemic districts; and aimed for total malaria-free districts by 2030 [4].

However, in contrast to this trend, a 23% increase in cases was observed between 2021 (1.5 million) and 2022 (1.8 million) [5]. Several nonexclusive factors may be responsible for this upsurge, including the spread of the Asian vector *Anopheles stephensi*, emergence and spread of drug-resistant and diagnostic-resistant *Plasmodium falciparum* parasites [6–9]. In addition, factors such as internal conflict and population displacement [5], climatic anomalies [10], and the COVID-19 pandemic [3, 11, 12] are potential contributing factors to the increase in malaria cases.

The scaling-up of infection prevention, mainly through long-lasting insecticidal nets (LLINs) and indoor residual spraying (IRS), has played a significant role in fighting the burden of malaria [13, 14]. Changes in the biting behaviour (outdoor host-seeking behaviours) of vectors [15–17] and increased insecticide resistance [18–21] have led to a renewed interest in larval source management (LSM) as a supplement to the main vector control interventions [22, 23]. Historically, LSM was part of the programmes to suppress malaria in the United States, Italy, and Israel [24, 25], and an essential intervention in eliminating *Anopheles arabiensis*, the African malaria vector, from Brazil [25]. Larvicide may be of particular value in urban areas [26, 27] and when associated with large-scale construction projects in malaria-endemic areas [28].

A pilot LSM programme in Equatorial Guinea that used *Bacillus thuringiensis israelensis* (*Bti*) was found to be an effective intervention tool when it was incorporated into vector-control strategies targeting large-scale construction projects, although more work on multisectoral coordination, associated costs, and changes in government policies was required for its effectiveness and sustainability [28]. In addition, a recent report showed that *Bti* is a promising tool for significantly reducing the incidence of malaria when combined with LLINs [29].

A previous four-arm trial (control, community-based house improvement, LSM, and community-based house improvement plus LSM) assessed the relative contribution of the interventions in the context of high insecticide-treated bed net coverage in Malawi. However, they found that LSM and housing improvement either alone or in combination did not further reduce malaria transmission or prevalence beyond the level reached in the control arm [30]. In 2022, the PMI Vector Link Project in Ethiopia implemented LSM in eight towns where the presence of *An. stephensi* was confirmed, including Awash, Semera-Logia, Batu, Meki, Dire Dawa, Degehabur, Godey, and Kebridehar, to evaluate larval density, habitat indices, and species composition.

The indicators used to measure the reduction in the immature mosquito population in the LSM intervention sites were mean larval density/20 dips, larval positivity of habitats, pupal density, and pupal positivity of habitats sustained for five consecutive months (from November 2022 to April 2023) [31]. A study that assessed breeding habitats in eastern Ethiopia suggested that LSM could provide an opportunity for focused control of *An. stephensi* [32]. However, there are no studies on the effectiveness of LSMs in reducing the incidence/prevalence of malaria in Ethiopia. Hence, this study opted to bridge this vital gap by modelling the trends of malaria cases based on risk rates before and after the LSM intervention and accounting for seasonal variability in Dire Dawa and Batu towns using Metehara town as a control group.

## Methods

### Study setting, design, and data description

Supported by the President’s Malaria Initiative (PMI) Vector Link Project, LSM was implemented in eight selected towns and cities where the presence of *An. stephensi* was confirmed in August 2022. This study assessed two LSM intervention sites (Dire Dawa city administration and Batu town) and a control town, Metehara which is also infected by *An. stephensi* and has similar weather conditions. The selection of the study area was based on the longer duration of LSM intervention implementation. While complete coverage was claimed in Batu town, in the Dire Dawa city administration among nine woredas, three malaria hotspot districts/woredas (Gende Kore, Sabian, and Melka Jebdu) were targeted.

**Fig. 1:**
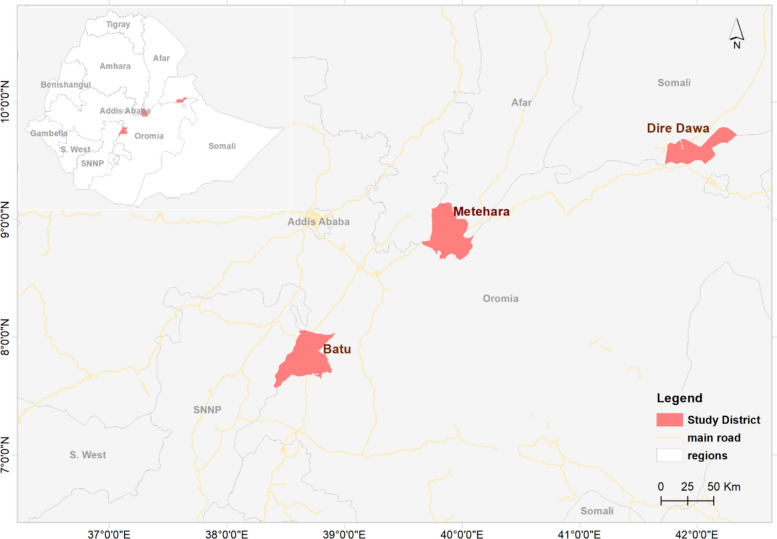
Study settings: Dire Dawa, Metehara, and Batu

The Dire Dawa city administration is located at 9° 36′ N 41° 52′ E along the Ethio-Djibouti transportation corridor. Dire Dawa is an eastern free trade zone with an estimated 550,642 residents. The selected woreda accounted for 32% (176,416/550, 642) of the total population and was the epicenter for the recent outbreak of malaria associated with *An. stephensi* [6]. Batu town is located at 7° 56′ N and 38° 42′ E, with an estimated total population of 84,595. In both Dire Dawa city administration and Batu town, the LSM was conducted along with the existing IRS and LLINs vector control approaches (Fig. 1).

**Fig. 2:**
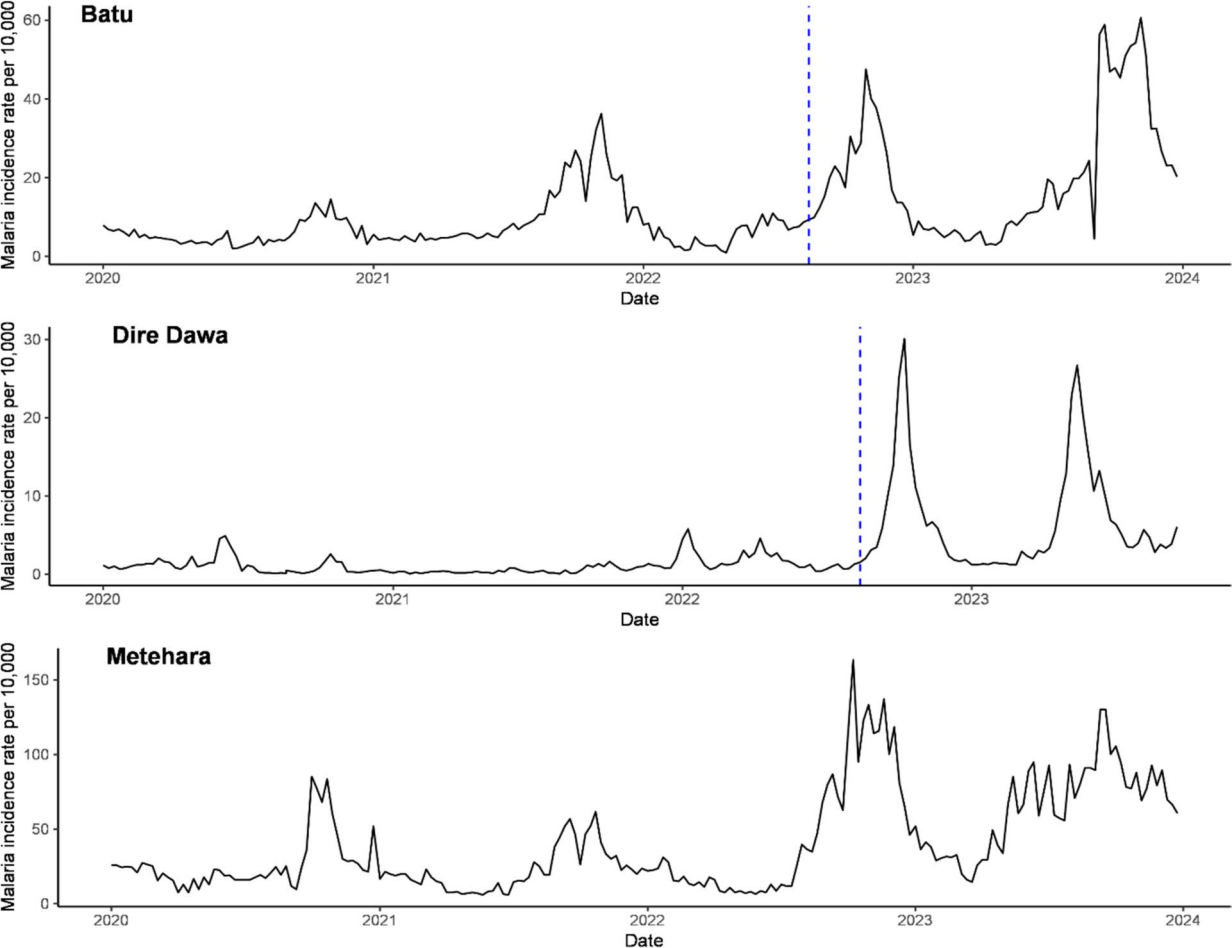
The temporal patterns of malaria incidence rate before and after intervention per 10,000 population. The broken vertical line represents the beginning of the intervention

Weekly confirmed malaria cases and population data were obtained from the city administration for Dire Dawa and the Oromia Regional State Health Bureau for Batu and Metehara towns (Table 1). Therefore, a controlled interrupted time series study was conducted to assess the effectiveness of the LSM on the weekly malaria incidence rate from mid-August 2022 to the end of 2023. In some weeks, the number of confirmed clinical cases was not reported (2% missing in Dire Dawa, 10% in Batu, and 7% in Metehara). A weighted moving average was employed to impute these missing numbers. This was done by averaging the data from the three weeks before and the three weeks after the missing week, all from the same town. Figure 2 presents the weekly malaria incidence rate per 10,000 people in each of the selected towns, along with the time of intervention, marked by a dashed vertical line.

**Table 1:** Population size of the study setting used to compute the risk rate over time

Year	Study setting		
	Dire Dawa	Batu	Metehara
2020	162,318	70,958	37,801
2021	166,919	74,692	38,970
2022	171,624	78,624	40,175
2023	176,416	82,762	41,418

### Larval source management implementation strategies

The PMI team sustained LSM with coverage of 90,000 individual larval habitats using either *Bti*-based larvicide application or source reduction every two weeks for up to 11 months. The LSM intervention was launched in August 2022 in both Dire Dawa and Batu. The targets of the project bi-weekly larviciding and larval source reduction and monitoring across the intervention sites were completed [31].

### Study variables

The dependent variable was the logarithm of the malaria incidence rate, while the independent variables were time, the presence or absence of the LSM intervention, time since the intervention, and the epidemic week.

### Data processing and statistical analysis

The weekly number of malaria cases denoted as $\:{y}_{t}$, and the exposed population, denoted as $\:{P}_{t}$, at any given week $\:t\:$ in each intervention or control site were used to compute the weekly rate of malaria cases per 10,000 population, *r*_t_: using the population size indicated in Table 1.

$$\:{r}_{t}=\text{10,000}\times\:\frac{{y}_{t}}{{P}_{t}}.$$

To tackle the common issue of right skewness in the rates, we applied a logarithmic transformation defined as $\:{v}_{t}=\text{log}\left({r}_{t}\right)$. This transformation results in transformed malaria incidence rates that have a more symmetric distribution.

The interrupted time series analysis framework assumed that the weekly rate of malaria incidence cases at each site followed the model.

$$\:{v}_{t}=\alpha\:+{\beta\:}_{1}t+{\beta\:}_{2}{x}_{t}+{\beta\:}_{3}{z}_{t}+{s}_{t}+{\epsilon}_{t}$$

with the terms of the model defined as:

- $\:\alpha\:$ represents the baseline level.
- $\:{\beta\:}_{1}$ is the coefficient of an overall linear temporal trend.
- $\:{x}_{t}$ is the dummy variable of the intervention status, where $\:{x}_{t}=0$ for weeks $\:t\:$ before the intervention and $\:{x}_{t}=1$ for weeks $\:t\:$ after the intervention.
- $\:{\beta\:}_{2}$ represents the level of change due to the immediate impact of the intervention.
- $\:{z}_{t}$ is the dummy variable indicating the number of weeks passed since the intervention occurred, where $\:{z}_{t}=0$ for weeks $\:t\:$ before the intervention.
- $\:{\beta\:}_{3}$ represents the trend change due to the lasting impact of the intervention.
- $\:{s}_{t}$ represents random periodic seasonal variations, following a cyclic second-order random walk structure.
- $\:{\epsilon}_{t}$ denotes unstructured random errors due to any remaining sources of variation.

To validate the biologically feasible two-week delay between the period before intervention and the anticipated onset of observable effects. The non-parametric Cox and Stuart trend test was used [33] to identify significant changes in the trend of the logarithm of malaria incidence rates. In addition, the Chow test was utilized to determine the most likely trend changes in these logarithmic rates by segmenting the data. This segmentation was conducted to assess differences in relationships between the segments, ensuring that each segment contained at least 20% of the observations [34].

After exploratory data analysis and nonparametric tests, an interrupted time series analysis was employed considering a periodic seasonal term with a cyclic second-order random walk structure to better account for seasonal variations in the data. In addition, since the data contain missing values moving averages imputation technique was used to impute these missing values. All the computations for this approach were implemented through the Integrated nested Laplace approximation (INLA) of the R package [35, 36].

## Results

### Temporal patterns of incidence of malaria and risk rate

A trend plot of clinically confirmed malaria incidence at two intervention sites (Batu and Dire Dawa) and the control site (Metahara) during the study period is illustrated in Fig. 2. An overall increasing pattern in the weekly clinically confirmed malaria incidence was noted at both the intervention and control sites before and after the intervention. The temporal patterns of risk rates before and after the intervention per 10,000 population illustrated that there was no visible decline in the risk rate of weekly confirmed malaria cases observed in any of the settings.

The Cox and Stuart trend test showed a significant change in the trend of the logarithm of malaria incidence rates in all three towns. The *p*-values, all below 0.001 for the three selected sites, indicated a significant shift in the trends. This result supports the use of trend change modelling approaches, such as the interrupted time series method. Furthermore, the result from change detection identifies suggested three-time points for Batu (“2020-12-16”, “2022-01-08”, “2022-12-24”), and Metahara (“2020-10-07”, “2021-07-23”, “2022-07-16”) and one-time point for Dire Dawa (“2021-08-20”) the most likely trend changes in the logarithm of malaria rates by segmenting the data.

### Modelling the effectiveness of the LSM

Table 2 presents the posterior mean and 95% credible intervals for the interrupted time series trend terms in the model: the overall trend, the level change due to the immediate impact of the intervention, and the trend change due to the lasting impact of the intervention. The model fitted for Dire Dawa revealed the overall trend is significantly increasing (positive slope) Dire Dawa shows a significant immediate level change. Similarly, the model fitted for Batu showed a significant increase in overall trend. However, in the model fitted for the control site Metehara, there was a significant decrease (negative slope) in the overall trend, although all three with very gradual slopes. Interestingly, despite no intervention in Metehara, there is a significant level and trend change after the intervention time, with the trend changing from decreasing to increasing.

**Table 2:** Posterior means and 95% credible intervals for the overall trend, the level change (immediate impact of the intervention), and the trend change (lasting impact of the intervention) for the three selected sites

Towns	Model coefficient	Mean	95% credible interval	Sig.
Dire Dawa	Overall (linear) trend	0.004	(0.000, 0.007)	*
	Level change (Immediate impact)	1.462	(0.891, 2.035)	*
	Trend change (Lasting impact)	0.003	(− 0.012, 0.018)	
Batu	Overall (linear) trend	0.003	(0.001, 0.005)	*
	Level change (Immediate impact)	0.007	(− 0.235, 0.249)	
	Trend change (Lasting impact)	0.008	(0.003, 0.013)	*
Metehara	Overall (linear) trend	− 0.002	(− 0.004, − 0.001)	*
	Level change (Immediate impact)	1.087	(0.865, 1.310)	*
	Trend change (Lasting impact)	0.006	(0.001, 0.010)	*

Significant terms are marked with a star in the Sig. Column

**Fig. 3:**
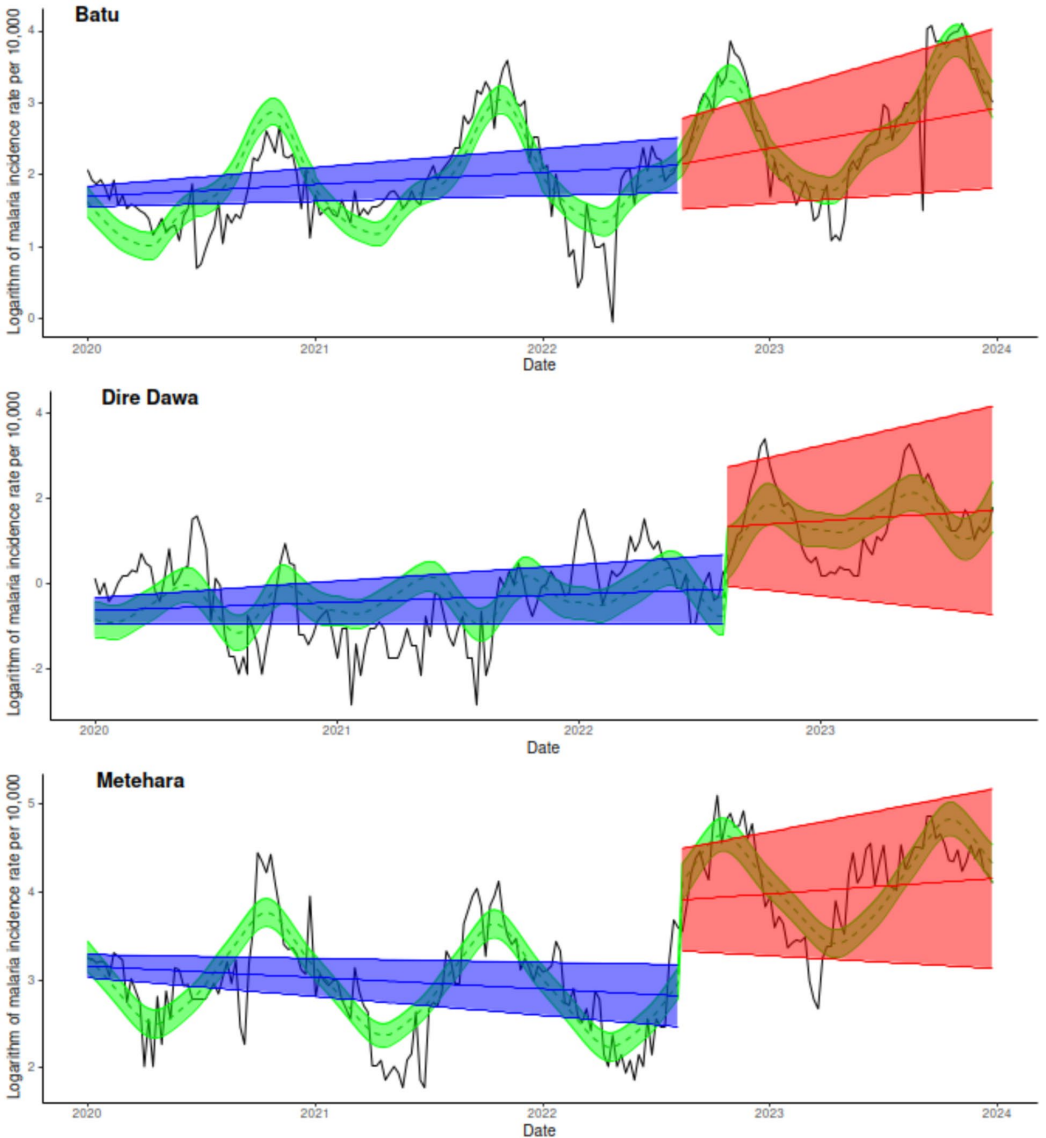
Posterior means and 95% credible intervals of the interrupted trends before (blue) and after (red) the intervention, along with the seasonal term (green), for the three selected sites

To further explore these findings, Fig. 3 illustrates the posterior mean and 95% credible intervals for the interrupted trends before (blue) and after (red) the intervention across all three sites. Notably, Metehara, the control site, experienced a significant trend change after the intervention. In Dire Dawa, the level of the trend shifted after the intervention, but the slope remained unchanged, while Batu saw a slight increase in the slope. For completeness, Fig. 3 also includes the posterior mean and 95% credible intervals for the seasonal term (green).

## Discussion

This study measured the impact of the temporal effectiveness of LSM as a vector control strategy via assessing weekly confirmed malaria case trends from 2020 to 2023 in intervention and control settings in Ethiopia. An overall increasing trend in the malaria incidence rate was observed irrespective of the implementation of LSM. The malaria incidence rate in these settings was characterized by seasonal fluctuations with significant increases after 2020. The immediate impact of the intervention and the trend change due to the lasting impact of the intervention Posterior means coefficient of intervention sites Dire Dawa and Batu revealed that there was a significant increasing malaria incidence rate obtained before and after the intervention. Others have argued that despite the extensive advocacy for LSM, it made no palpable contribution to the achievements in malaria reduction [37]. The increased malaria incidence rate of weekly confirmed malaria cases after the intervention indicates the presence of productive breeding habitats and/or potential contamination, as reported elsewhere [30, 38].

However, the observed increase in confirmed malaria incidence rate after 2020 at the study sites is in line with the overall nationwide increase in malaria incidence rate since 2019 [5, 39–41]. The increase in malaria incidence rate is attributed to the COVID-19 pandemic [1, 42], diagnostic and drug-resistant *Plasmodium* parasites [6–9], and the emergence of *An. stephensi* [6, 7], climate change [10] and internal conflicts and population displacements [43, 44].

The observed lack of impact of LSM is consistent with what has been reported previously, and the efficacy and residual activity of *Bti*-based application on malaria vectors could be influenced by factors such as mosquito species, mosquito development period, larval habitat conditions, and larvicide properties [45–47]. Some authors have claimed that LSM is unfeasible in African transmission settings due to the high number of small and temporary larval habitats for *An. gambiae* that are difficult to find and treat promptly [48] and only work best in areas where larval habitats can be well defined [25]. Larvicides have limitations in tropical African settings, and careful testing under field conditions is needed before they can be used for malaria vector control. For instance, higher temperatures increase larvicide efficacy, partly due to an increased larval feeding rate [49, 50]. Thus, the inherent difference in its activity in variable ecological settings needs to be considered when implementing this tool.

After extensive one-year breeding site mapping, LSM intervention in Dar es Salam resulted in a 40% overall reduction in *Plasmodium* parasite prevalence, with the highest impact achieved in the dry season [51]. Another study revealed that larvicide intervention is most effective in reducing malaria transmission during the dry season; individuals living in areas where larvicide was not implemented exhibited 21% higher malaria infection rates and showed protective effects in combination with insecticide-treated bed nets. They also observed no evidence of spillover effects between intervention and control sites [52]. In addition, other studies in Tanzania showed that the LSM strategy reduced both the densities of target mosquitoes and the prevalence of malaria [53, 54]. Furthermore, a Cochrane Review on LSM showed a 75% reduction in *Plasmodium* parasite prevalence and a 69% drop in incidence in some settings [55]. Curiously, despite the sustained reduction in the indices, the mean larval density/20 dips, mean larval positivity of habitats, mean pupal density and mean pupal positivity of habitats for consecutive months from November 2022 to April 2023 [31], the entomological result did not translate to a reduction in the malaria incidence rate (Fig. 3).

The limitations of this study are related to the underreported malaria case counts, as the data are retrospective. In addition, this study does not account for the effects of climate and environmental factors that may have reduced the effectiveness of LSMs. Only three sites were targeted for LSM at Dire Dawa city administration, there might be potential contamination and repopulation of breeding habitats from other nearby sites without LSM intervention.

## Conclusion

In conclusion, although LSM was effective in reducing larval indices, as indicated by the PMI Vector Link Report [31], it was not translated into reducing the malaria incidence rate. Thus, LSM intervention implementation needs to be reviewed to tailor to specific settings and/or assess the suitability of the intervention before large-scale implementation.

## Data Availability

All data generated or analyzed during this study are included in the manuscript.

## Abbreviations

LSM: Larval Source Management
PMI: President’s Malaria Initiative
SARIMA: Seasonal Autoregressive Integrated Moving Average
ITS: Interrupted Time Series
PHEM: Public Health Emergency Management

## References

[1] WHO. World malaria report 2020: 20 years of global progress and challenges. Geneva: World Health Organization; 2021.

[2] Deribew A, Dejene T, Kebede B, Tessema GA, Melaku YA, Misganaw A, et al. Incidence, prevalence and mortality rates of malaria in Ethiopia from 1990 to 2015: analysis of the global burden of diseases 2015. Malar J. 2017;16:271. 28676108 10.1186/s12936-017-1919-4 PMC5496144

[3] NMEP. Ethiopia Malaria Elimination Strategic Plan: 2021–2025. Addis Ababa: Ministry of Health; 2020.

[4] NMER. National Malaria Elimination Roadmap: 2017–2030. Addis Ababa: Ministry of Health; 2021.

[5] WHO. World Malaria Report 2023. Geneva: World Health Organization; 2023.

[6] Emiru T, Getachew D, Murphy M, Sedda L, Ejigu LA, Bulto MG, et al. Evidence for a role of *Anopheles Stephensi* in the spread of drug and diagnosis-resistant malaria in Africa. Nat Med. 2023;29:3203–11. 37884028 10.1038/s41591-023-02641-9 PMC10719088

[7] Fola AA, Feleke SM, Mohammed H, Brhane BG, Hennelly CM, Assefa A et al. Clonal spread of *Plasmodium falciparum* candidate artemisinin partial resistance Kelch13 622I mutation and co-occurrence with pfhrp2/3 deletions in Ethiopia. medRxiv. 2023 (preprint).

[8] Mihreteab S, Platon L, Berhane A, Stokes BH, Warsame M, Campagne P, et al. Increasing prevalence of artemisinin-resistant HRP2-negative malaria in Eritrea. N Engl J Med. 2023;389:1191–202. 37754284 10.1056/NEJMoa2210956 PMC10539021

[9] Assefa A, Fola AA, Tasew G. Emergence of *Plasmodium falciparum* strains with artemisinin partial resistance in East Africa and the Horn of Africa: is there a need to panic? Malar J. 2024;23:34. 38273360 10.1186/s12936-024-04848-8 PMC10809756

[10] Woyessa A, Siebert A, Owusu A, Cousin R, Dinku T, Thomson MC. El Niño and other climatic drivers of epidemic malaria in Ethiopia: new tools for national health adaptation plans. Malar J. 2023;22:195. 37355627 10.1186/s12936-023-04621-3 PMC10290321

[11] Daba C, Atamo A, Debela SA, Kebede E, Woretaw L, Gebretsadik D, et al. A retrospective study on the burden of malaria in Northeastern Ethiopia from 2015 to 2020: implications for pandemic preparedness. Infect Drug Resist. 2023;16:821–8. 36818806 10.2147/IDR.S399834 PMC9930572

[12] Weiss DJ, Bertozzi-Villa A, Rumisha SF, Amratia P, Arambepola R, Battle KE, et al. Indirect effects of the COVID-19 pandemic on malaria intervention coverage, morbidity, and mortality in Africa: a geospatial modelling analysis. Lancet Infect Dis. 2021;21:59–69. 32971006 10.1016/S1473-3099(20)30700-3 PMC7505634

[13] WHO. Global insecticide use for vector-borne disease control: a 10-year assessment [‎2000–2009]‎. 5th ed. Geneva: World Health Organization; 2011.

[14] Bhatt S, Weiss DJ, Cameron E, Bisanzio D, Mappin B, Dalrymple U, et al. The effect of malaria control on *Plasmodium Falciparum* in Africa between 2000 and 2015. Nature. 2015;526:207–11. 26375008 10.1038/nature15535 PMC4820050

[15] Bedasso AH, Gutto AA, Waldetensai A, Eukubay A, Bokore GE, Kinde S, et al. Malaria vector feeding, peak biting time and resting place preference behaviors in line with indoor based intervention tools and its implication: scenario from selected sentinel sites of Ethiopia. Heliyon. 2022;8:e12178. 36578426 10.1016/j.heliyon.2022.e12178 PMC9791363

[16] Kibret S, Wilson G. Increased outdoor biting tendency of *Anopheles arabiensis* and its challenge for malaria control in Central Ethiopia. Public Health. 2016;141:143–5. 27931990 10.1016/j.puhe.2016.09.012

[17] Degefa T, Githeko AK, Lee M-C, Yan G, Yewhalaw D. Patterns of human exposure to early evening and outdoor biting mosquitoes and residual malaria transmission in Ethiopia. Acta Trop. 2021;216:105837. 33485868 10.1016/j.actatropica.2021.105837 PMC8682696

[18] Demissew A, Animut A, Kibret S, Tsegaye A, Hawaria D, Degefa T, et al. Evidence of pyrethroid resistance in *Anopheles Amharicus* and *Anopheles arabiensis* from Arjo-Didessa irrigation scheme, Ethiopia. PLoS ONE. 2022;17:e0261713. 35030201 10.1371/journal.pone.0261713 PMC8759678

[19] Yared S, Gebressielasie A, Damodaran L, Bonnell V, Lopez K, Janies D, et al. Insecticide resistance in *Anopheles stephens*i in Somali Region, eastern Ethiopia. Malar J. 2020;19:180. 32398055 10.1186/s12936-020-03252-2 PMC7216317

[20] Mekuriaw W, Yewhalaw D, Woyessa A, Bashaye S, Massebo F. Distribution and trends of insecticide resistance in malaria vectors in Ethiopia (1986–2017): a review. Ethiop K Public Health Nutr. 2019;3:51–61.

[21] Alemayehu E, Asale A, Eba K, Getahun K, Tushune K, Bryon A, et al. Mapping insecticide resistance and characterization of resistance mechanisms in *Anopheles arabiensis* (Diptera: Culicidae) in Ethiopia. Parasit Vectors. 2017;10:407. 28865490 10.1186/s13071-017-2342-y PMC5581456

[22] Tusting LS, Thwing J, Sinclair D, Fillinger U, Gimnig J, Bonner KE, et al. Mosquito larval source management for controlling malaria. Cochrane Database Syst Rev. 2013;2013:CD008923. 23986463 10.1002/14651858.CD008923.pub2 PMC4669681

[23] Meyers JI, Pathikonda S, Popkin-Hall ZR, Medeiros MC, Fuseini G, Matias A, et al. Increasing outdoor host-seeking in *Anopheles gambiae* over 6 years of vector control on Bioko Island. Malar J. 2016;15:239. 27113244 10.1186/s12936-016-1286-6 PMC4845310

[24] Kitron U, Spielman A. Suppression of transmission of malaria through source reduction: antianopheline measures applied in Israel, the United States, and Italy. Rev Infect Dis. 1989;11:391–406. 2665000 10.1093/clinids/11.3.391

[25] Fillinger U, Lindsay SW. Larval source management for malaria control in Africa: myths and reality. Malar J. 2011;10:353. 22166144 10.1186/1475-2875-10-353 PMC3273449

[26] Antonio-Nkondjio C, Sandjo NN, Awono-Ambene P, Wondji CS. Implementing a larviciding efficacy or effectiveness control intervention against malaria vectors: key parameters for success. Parasit Vectors. 2018;11:57. 29368633 10.1186/s13071-018-2627-9 PMC5784718

[27] WHO. Larval source management: a supplementary malaria vector control measure: an operational manual. Geneva: World Health Organization; 2013.

[28] García G, Fuseini G, Donfack OT, Wofford RN, Nlang JAM, Efiri PB, et al. The need for larval source management accompanying urban development projects in malaria endemic areas: a case study on Bioko Island. Malar J. 2022;21:328. 36376966 10.1186/s12936-022-04362-9 PMC9664620

[29] Tia J-PB, Tchicaya ES, Zahouli JZ, Ouattara AF, Vavassori L, Assamoi J-B, et al. Combined use of long-lasting insecticidal nets and *Bacillus thuringiensis* israelensis larviciding, a promising integrated approach against malaria transmission in northern Côte d’Ivoire. Malar J. 2024;23:168. 38812003 10.1186/s12936-024-04953-8 PMC11137964

[30] McCann RS, Kabaghe AN, Moraga P, Gowelo S, Mburu MM, Tizifa T, et al. The effect of community-driven larval source management and house improvement on malaria transmission when added to the standard malaria control strategies in Malawi: a cluster-randomized controlled trial. Malar J. 2021;20:232. 34022912 10.1186/s12936-021-03769-0 PMC8140568

[31] The PMI VectorLink Project. Ethiopia Final Entomological Report, April 2022-March 2023. Rockville: Abt Associates; 2023.

[32] Yared S, Gebresilassie A, Aklilu E, Abdulahi E, Kirstein OD, Gonzalez-Olvera G, et al. Building the vector in: construction practices and the invasion and persistence of *Anopheles stephensi* in Jigjiga, Ethiopia. Lancet Planet Health. 2023;7:e999–1005. 38056970 10.1016/S2542-5196(23)00250-4 PMC11707895

[33] Sprent P, Smeeton NC. Applied nonparametric statistical methods. Boca Raton: CRC Press; 2016.

[34] Zeileis A, Shah A, Patnaik I. Testing, monitoring, and dating structural changes in exchange rate regimes. Comput Stat Data Anal. 2010;54:1696–706.

[35] Wang X, Yue YR, Faraway JJ. Bayesian regression modeling with INLA. Boca Raton: Chapman and Hall/CRC; 2018.

[36] Martins TG, Simpson D, Lindgren F, Rue H. Bayesian computing with INLA: new features. Comput Stat Data Anal. 2013;67:68–83.

[37] Newman RD, Mnzava A, Szilagyi Z. Mosquito larval source management: evaluating evidence in the context of practice and policy. Cochrane Database Syst Rev. 2013;2013:ED000066. 24156097 10.1002/14651858.ED000066 PMC10846359

[38] McCann RS, van den Berg H, Takken W, Chetwynd AG, Giorgi E, Terlouw DJ, et al. Reducing contamination risk in cluster-randomized infectious disease-intervention trials. Int J Epidemiol. 2018;47:2015–24. 30376050 10.1093/ije/dyy213

[39] Nigussie TZ, Zewotir TT, Muluneh EK. Seasonal and spatial variations of malaria transmissions in northwest Ethiopia: evaluating climate and environmental effects using generalized additive model. Heliyon. 2023;9:e15252. 37089331 10.1016/j.heliyon.2023.e15252 PMC10114238

[40] Olani Z, Solomon S, Kaba Z, Bikila H. A five-year (2016–2020) trend analysis of malaria surveillance data in Oromia Regional State, Ethiopia. Biomed Res Int. 2023;2023:5278839. 37576999 10.1155/2023/5278839 PMC10423085

[41] Teka H, Golassa L, Medhin G, Balkew M, Sisay C, Gadisa E, et al. Trend analysis of malaria in urban settings in Ethiopia from 2014 to 2019. Malar J. 2023;22:235. 37580690 10.1186/s12936-023-04656-6 PMC10426206

[42] Hakizimana D, Ntizimira C, Mbituyumuremyi A, Hakizimana E, Mahmoud H, Birindabagabo P, et al. The impact of Covid-19 on malaria services in three high endemic districts in Rwanda: a mixed-method study. Malar J. 2022;21:48. 35164781 10.1186/s12936-022-04071-3 PMC8845295

[43] Debash H, Nigatie M, Bisetegn H, Feleke DG, Tesfaw G, Amha A, et al. Malaria surveillance, outbreak investigation, response and its determinant factors in Waghemra Zone, Northeast Ethiopia: unmatched case–control study. Sci Rep. 2023;13:9938. 37336906 10.1038/s41598-023-36918-3 PMC10279665

[44] Yu Q, Qu Y, Zhang L, Yao X, Yang J, Chen S, et al. Spatial spillovers of violent conflict amplify the impacts of climate variability on malaria risk in sub-Saharan Africa. Proc Natl Acad Sci USA. 2024;121:e2309087121. 38557184 10.1073/pnas.2309087121 PMC11009658

[45] Lacey LA. Bacillus thuringiensis serovariety israelensis and *Bacillus sphaericus* for mosquito control. J Am Mosq Control Assoc. 2007;23:133–63. 17853604 10.2987/8756-971X(2007)23[133:BTSIAB]2.0.CO;2

[46] Dambach P, Louis VR, Kaiser A, Ouedraogo S, Sié A, Sauerborn R, et al. Efficacy of *Bacillus thuringiensis* var. israelensis against malaria mosquitoes in northwestern Burkina Faso. Parasit Vectors. 2014;7:371. 25128297 10.1186/1756-3305-7-371 PMC4262221

[47] Derua YA, Tungu PK, Malima RC, Mwingira V, Kimambo AG, Batengana BM, et al. Laboratory and semi-field evaluation of the efficacy of *Bacillus thuringiensis* var. israelensis (Bactivec^®^) and *Bacillus sphaericus* (Griselesf^®^) for control of mosquito vectors in northeastern Tanzania. Curr Res Parasitol Vector Borne Dis. 2022;2:100089. 35664894 10.1016/j.crpvbd.2022.100089 PMC9157456

[48] Najera A, Zaim M. Malaria vector control: decision making criteria and procedures for judicious use of insecticides. Geneva: World Health Organization; 2003.

[49] Mittal P, Adak T, Sharma V. Effect of temperature on toxicity of two bioinsecticides spherix *(Bacillus sphaericus*) and bactoculicide (*Bacillus thuringiensis*) against larvae of four vector mosquitoes. Indian J Malariol. 1993;3:37–41. 8100540

[50] Rasnitsyn S, Voĭtsik A, Iasiukevich V. The effect of water temperature on the action of bacterial insecticides against mosquito larvae (in Russian). Med Parazitol (Mosk). 1993:8–10. 8336659

[51] Fillinger U, Kannady K, William G, Vanek MJ, Dongus S, Nyika D, et al. A tool box for operational mosquito larval control: preliminary results and early lessons from the Urban Malaria Control Programme in Dar es Salaam, Tanzania. Malar J. 2008;7:20. 18218148 10.1186/1475-2875-7-20 PMC2259364

[52] Maheu-Giroux M, Castro MC. Impact of community-based larviciding on the prevalence of malaria infection in Dar es Salaam, Tanzania. PLoS ONE. 2013;8:e71638. 23977099 10.1371/journal.pone.0071638 PMC3743749

[53] Mwakalinga VM, Sartorius BK, Limwagu AJ, Mlacha YP, Msellemu DF, Chaki PP, et al. Topographic mapping of the interfaces between human and aquatic mosquito habitats to enable barrier targeting of interventions against malaria vectors. R Soc Open Sci. 2018;5:161055. 29892341 10.1098/rsos.161055 PMC5990771

[54] Msellemu D, Namango HI, Mwakalinga VM, Ntamatungiro AJ, Mlacha Y, Mtema ZJ, et al. The epidemiology of residual *Plasmodium falciparum* malaria transmission and infection burden in an African city with high coverage of multiple vector control measures. Malar J. 2016;15:288. 27216734 10.1186/s12936-016-1340-4 PMC4877954

[55] RBM-Vector-Control-Working-Group. 3rd Larval Source Management Work Stream Meeting. Geneva: RBM; 201.

